# On Cleft Palate, and on Staphyloraphy

**Published:** 1848-01

**Authors:** William Fergusson

**Affiliations:** Professor of Surgery in King's College London.


### ARTICLE XI.

*On Cleft Palate, and on Staphyloraphy.* By WILLIAM FERGUSSON, Esq., F. R. S. E., Professor of Surgery in King's College London.

From the Medical Times.

*Author's experience increased since the reading of his paper on these subjects in* 1844, *before the Medical and Chirurgical Society of London; nature of cleft palate; different forms and kinds of; its complication with hare-lip; effects of malformation on deglutition and on voice and speech; method of obviating defects before modern operation of staphyloraphy; advantages and disadvantages of latter proceeding; indifferent success of the operation, although performed by most of the leading surgeons in Europe and America; success of Roux, Mütter, and Dr. J. M. Warren; apathy of British surgeons on the subject; author's investigations; anatomy and physiology of cleft palate first described by him; new operation proposed and performed by author, and its success in various instances; reviews of different proposals, by Roux, Bushe, Dieffenbach, Pancoast, Liston, Mittauer, and J. M. Warren; fit cases for operation; age proper for its performance; author's operation described; ligatures and knots; new knot; after treatment; causes of failure of operation; examples of success of author's plan; effects of operation on voice and speech, &c.*

Two years have now elapsed since I delivered a special lecture on these subjects, and about the same time I communicated a paper to the Royal Medical and Chirurgical Society, which was afterwards honored with a place in the volume of "Transactions" for the year 1845. During the interval my experience has accumulated, and I am now desirous of stating the results, while, at the same time, I bring before my pupils as much upon such topics as pertains to the duties of the chair which I occupy in this college.

The state of the mouth, familiarly known under the name of cleft palate, is congenital, although a condition somewhat analogous to it occasionally results from injury or disease. It is chiefly to the natural malformation that the following observations apply, although they are suitable in many respects to other imperfections in the palate and roof of the mouth generally. The congenital defect is met with in a variety of forms: in some instances the fissure is limited to the uvula, when that part appears double; in others the fissure extends further forward, so as to leave the velum in two halves; the whole, or a portion only of this part, may be thus; the fissure may extend partially or entirely through the hard palate; the alveolar ridge may also be implicated, in which case the condition is usually, if not always, accompanied with fissure in the upper lip as well. The hare-lip and cleft palate very frequently go together, although it is by no means unusual to see either the one or the other by itself. The fissure in the uvula and soft palate is invariably in the mesial line; even in the hard palate it appears to be so, although commonly the vomer is attached below, directly in the middle, and the fissure communicates with one nostril only. When the alveoli are divided also, the fissure is always to the side, and this is particularly conspicuous when there is a double hare-lip present at the same time. There is, then, an intermaxillary bone immediately underneath the columna, on which rests the middle portion of the upper lip. Sometimes the fissure appears as a narrow slit, and in other instances is so wide and large as to cause the mouth and nostril to appear like one huge cavity.

The effects of cleft palate are observable in various ways. In infancy, if the malformation be at all extensive, there may be imperfect nourishment in consequence of part of the food passing into the nostrils during deglutition; and in advanced years the peculiar tone of voice and defective pronunciation are remarkably characteristic of this condition. Infants sometimes die from inanition in this state; and in the adult the deficiencies alluded to are often such as to mar his prospects in life. The tone of the voice often gives rise to unjust suspicions against the sufferer, and there are many so conscious of the unpleasant sound that they use it as little as possible.

Until the present century, the means known for obviating the defects alluded to were of a very imperfect kind. In the adult, the opening could be closed only by an obturator or false palate; and in the child, even now, we know of no other remedy for the defect than a spoon or bottle, with some contrivance attached to it whereby the food is passed almost into the pharynx at once. In modern times, however, something more has been done for the adult; and it has been proposed to treat the fissure in the palate on the same principles as have long guided us in cases of hare-lip. Roux and Graefe in Europe, and Warren in America, pared the edges of the cleft, and brought them into contact in such a manner as to permit union by the first intention; and so the malformation was soon remedied, as in the more familiar example of hare-lip.

The intention with which an obturator or false palate is applied is, that, by closing the direct communication between the mouth and nostrils, the sound of the voice may pass outwards through the mouth without the nasal accompaniment which gives its unpleasant character. It is also used to prevent the escape of food upwards: for although the adult, by long custom and constant care, can generally swallow all his food and drink, yet in an unguarded moment, a lapse may occur, and cause much annoyance. The objects of the operation being the same as those just alluded to, it will be very apparent that, if the two sides of the palate can be brought together to have the same effects as the obturator, the advantages are great in favor of such a proceeding. If the operation be successful the parts are put in a condition similar in most respects, to their natural state, as if no such malformation had ever been present. As regards the voice and articulation, this was remarkably evinced in the case of the first patient on whom Roux operated. When the gentleman, a student of medicine, returned among his former friends, there was such a change for the better that he could not have been recognised by his speech alone.

Notwithstanding the apparent merits of the surgical operation for cleft palate (staphyloraphy, as it is called,) there are certain disadvantages connected with it which should always be borne in mind when an opinion is required on the propriety of performing an operation, or the probability of its results. Even with the utmost care the proceeding when done may end in partial or complete failure, there may be some portion of the fissure, perhaps in the hard palate, which may baffle the surgeon's skill; or, from causes over which he has no control, the union of the soft parts, which it is his object to effect, may not occur. Even under the most favorable circumstances, as regards union, the patient's speech may not be so much improved as had been anticipated. Then there are difficulties connected with the operation so vexatious, so trying to the surgeon's patience, as well as to the endurance of the patient himself, that it must always be looked upon as one of the most harassing that we are ever called upon to perform. The slightest opposition on the part of the patient may prevent the operation being performed; and a trifling indiscretion, in not attending to the injunctions of the surgeon afterwards, may mar the effect of the proceeding, however successfully it may have been accomplished at the time. All these circumstances, as observed in different cases, have, no doubt, had effect on the surgeon's mind, and as the successful results have not been commensurate with the seeming disadvantages have rendered him somewhat careless regarding the proceeding.

The operation has, nevertheless, been done by most of the leading surgeons of the day, among whom may be mentioned Roux, Graefe, the Warrens, father and son; Dieffenbach, Brodie, Guthrie, Liston, Bushe, Cusack, Crampton, Mittauer (of Virginia,) Mütter and Pancoast (of Philadelphia,) &c. In 1842 it was said that Roux had performed it upwards of one hundred times. In two-thirds of the simple cases, and in one-third of those which were complicated, the proceeding had been beneficial. In 1843, my friend Dr. Mütter had operated twenty-one times upon the soft and hard palate, and out of this number "had failed to relieve the patient but in two cases;"

and Dr. J. Mason Warren had been equally successful in thirteen out of fourteen instances, in which he had operated. Indeed the success of these gentlemen had seemingly been greater than that of the distinguished author of the operation—Roux, although it would be observed that the cases were far fewer in number than had been treated by that surgeon. Few though they are, however, comparatively, I do not suppose that any surgeon in this country has operated on so many; no statistics have been drawn; no one has had the courage to publish an account of his cases; and a general impression has prevailed that the proceeding has been very unsatisfactory. Among the various cases that had come under my own notice, until within a comparatively recent date, the constant failures had been such as to induce great apathy on my part towards the proceeding, an apathy which had been somewhat increased by the failure of an operation performed on a gentleman in London, by Roux himself, when here on a visit a few years ago; and also by a similar result following an operation done by Mr. Bowman, in whose hands the proceeding had received every justice. I mention the latter case thus pointedly, because I shall have to draw attention to it again in a future part of this lecture.

I had not, however, given up all interest in cases of this malformation, and certain circumstances induced me, about three years ago, to bestow more attention and study on the subject than I had done previously. A preparation of cleft palate in my possession gave me an opportunity of investigating the anatomy, and this led me to draw certain conclusions regarding the physiology and surgery of these parts, such as I believe had never been made before.

It had long been familiar to those accustomed to see such cases on the living body, that during deglutition the two portions of the uvula came together in the middle line; but no one had attempted to explain how this could happen, and even such an acute observer as M. Malgaigne stated, that it was "by a muscular action, of which it is difficult to give an explanation." The dissection of the parts enabled me to explain this in a way which I imagine is incontrovertible; and to show you how this happened, as well as for other purposes, it will now be best that I should explain to you the condition of the parts in this malformation, and contrast it in as far as may be requisite with the natural state.

The preparation now before you exhibits the upper part of the mouth and pharynx of an aged female subject. The muscles of the pharynx have been carefully dissected, as have also those connected specially with the palate. A glance at the roof of the mouth shows the gap in the mesial line, and how the uvula, soft palate, and a portion of the hard, are involved in the defect. Behind it may be observed that the constrictors are not so broad, so capacious, as in the natural condition, but that the muscular fibres are nevertheless as strongly developed. The upper border of the superior constrictor is especially well marked, and here it may be seen to form a kind of semicircular margin, extending between the basilar process of the occipital bone and the internal pterygoid plate, on which margin the levator palati muscle seems to rest. A perpendicular incision has been made through the pharynx behind, exactly in the mesial line, and, the mucous membrane having been stripped off the inside, the muscularity is thus rendered still more distinct. The mucous covering has also been taken off the upper surface of the palate, whereby one side of the nostril immediately above, and the muscles of the palate have been more extensively exposed.

It may now be seen how the two portions of the uvula and corresponding parts of the soft palate touch each other during deglutition, for it is evident that, as the superior constrictor muscles act, they must throw or push the soft tissues in front forwards and inwards; an effect which will be aided by the superior fibres of the middle constrictors, which, stretching across as they do from one side to the other, having no attachment mesially, as is also the case with the lower fibres of the superior muscles, must contribute powerfully to the result in question. A remarkable difference may here be observed between this and the normal state of the parts; the palato-pharyngei muscles are not attached to each other, as in the well-formed palate. These muscles are seen to form the principal part of the free margin of palate along the line of fissure; their course is somewhat semicircular from their upper end to their lower, the convexity being towards the middle; and it follows that during action, if not opposed in any way, they must pull the parts outwards, an action the reverse of that described by Dzondi, Müller, and others, as belonging to the muscles in their natural condition. The levator palati is seen throughout its entire course, and the tensor palati may also be clearly made out. The levator, it will be perceived, as I imitate its action by pulling it, not only acts very efficiently on the moveable portion of the palate, but its sphere of action, from the muscle being chiefly muscular throughout its entire course, is so great that, during rigid contraction, it must forcibly pull the soft parts upwards, backwards, and outwards. It is worthy of special observation, that the tensor or circumflexus palati has hardly any influence on the velum, for, pull as I choose upon it, there is only the slightest movement to be observed at the parts where its tendon spreads on the surface of the soft palate. Neither in the natural nor in the cleft palate can this muscle have a power at all to compare with the levator, which, from its length, position, and character generally, is the principal motor of this very mobile part. The anterior pillar of the fauces is very slight, and the fibres of the palato-glossus are indistinct; the posterior pillar, however, is distinct enough, and formed as in the natural state by the bundle of fibres of the palato-pharyngeus. The azygos uvulæ is by no means distinct; a bundle of fibres, about the size of a crowquill, may be seen on the lower part of each free margin of the soft palate.

From such an inspection as this preparation afforded, I was led to take those views of the physiology and surgery of the parts, the explanation of which forms the principal object of this lecture. It required no great foresight to perceive that the movement of each side of the palate must depend chiefly upon the action of the levator muscle and palato-pharyngeus. The influence of the levator muscle might have been calculated on from previous knowledge, but that of the palato-pharyngeus could scarcely have been thought of. Both must evidently have the effect of widening the fissure, especially the levator; and the various conditions under which the palate may be seen can be explained by reference to these two muscles. When the mouth is looked into, and the soft portions of the palate are in a quiescent state, the fissure will then appear probably in a medium state. A slight irritation, with a probe or point of the finger, will cause a corresponding movement—the soft parts will be drawn upwards and outwards, so that the gap will be enlarged. If the irritation be increased, the same parts will be acted on, that they will almost disappear on the sides of the fissure, but even now, if an effort at deglutition be made, the two portions of the uvula will be forced together, by the action of the superior constrictors, as already explained. It seems to me that under ordinary circumstances, after the operation for closing the fissure, the slightest irritation would be likely to call the levators and palato-pharyngei into action, and so induce that dragging on the stitches with which surgeons were so familiar—an influence sufficient, in some instances, to cause ulceration in the seat of the threads, or, in others, to cause separation of the recently-united parts. I therefore supposed that, if these muscles could be divided before bringing the edges of the palate together, the parts would remain so quiet immediately afterwards that there would be greater probability of union in the mesial line taking place than if the muscles were left entire or untouched. It was not long before I had an opportunity of testing the project on the living body. The result was so satisfactory that I tried it in another instance shortly afterwards, and here the effect was most complete. The two cases were appended to my paper on this subject when laid before the Royal Medico-Chirurgical Society, and since that date I have operated on eight more, making ten in all, in eight of which I have been perfectly successful in closing the soft palate. In some of these there has been fissure of the hard palate as well, and the parties have been content with the remaining comparatively small apertures, or have had them closed by obturators. I know of four other instances where the operation, conducted on the plan recommended by me, has been successful, and a fifth which failed. During the same period I have known three examples of failure by the ordinary method. Thus, out of fifteen cases on my plan, there have been three which did not succeed, while all those done in accordance with Roux's operation were failures.

(To be continued.)

---

### Miscellaneous Notices.

